# LncRNAs and PRC2: Coupled Partners in Embryonic Stem Cells

**DOI:** 10.3390/epigenomes3030014

**Published:** 2019-08-06

**Authors:** Alessandro Fiorenzano, Emilia Pascale, Eduardo Jorge Patriarca, Gabriella Minchiotti, Annalisa Fico

**Affiliations:** 1 Department of Experimental Medical Science and Lund Stem Cell Center BMC, Lund University, 22632 Lund, Sweden; 2 Stem Cell Fate Laboratory, Institute of Genetics and Biophysics “A. Buzzati-Traverso”, CNR, 80131 Naples, Italy; 3 Institute of Genetics and Biophysics “A. Buzzati-Traverso”, CNR, 80131 Naples, Italy

**Keywords:** embryonic stem cells, lncRNAs, PRC2, chromatin modification, single-cell analysis

## Abstract

The power of embryonic stem cells (ESCs) lies in their ability to self-renew and differentiate. Behind these two unique capabilities is a fine-tuned molecular network that shapes the genetic, epigenetic, and epitranscriptomic ESC plasticity. Although RNA has been shown to be functionally important in only a small minority of long non-coding RNA genes, a growing body of evidence has highlighted the pivotal and intricate role of lncRNAs in chromatin remodeling. Due to their multifaceted nature, lncRNAs interact with DNA, RNA, and proteins, and are emerging as new modulators of extensive gene expression programs through their participation in ESC-specific regulatory circuitries. Here, we review the tight cooperation between lncRNAs and Polycomb repressive complex 2 (PRC2), which is intimately involved in determining and maintaining the ESC epigenetic landscape. The lncRNA-PRC2 partnership is fundamental in securing the fully pluripotent state of ESCs, which must be primed to differentiate properly. We also reflect on the advantages brought to this field of research by the advent of single-cell analysis.

## 1. Introduction

A hallmark of embryonic stem cells (ESCs) is their ability to maintain a pluripotent and self-renewal network while retaining the capacity to promptly respond to a variety of microenvironmental signals activating the expression of gene differentiation programs [1,2]. ESC cellular plasticity is preserved by an intricate transcriptional regulatory circuitry involving the interplay between transcription factors (TFs), epigenetic machinery, and non-coding RNAs. Over the past two decades, our understanding of how the non-coding genome impacts on cell fate decisions and tissue patterning during development has vastly expanded [3,4,5]. Recent insights into long non-coding RNAs (lncRNAs) have challenged the conventional paradigm that RNA is merely a messenger passing information between DNA and protein, shifting the focus away from the protein-coding genome. A large portion of the mammalian genome encodes genes that transcribe lncRNAs without any apparent protein-coding potential [6,7]. Although little is yet known about the dynamic regulation of their transcription, biogenesis, and degradation, lncRNAs cannot be dismissed as transcriptional ‘noise’. Rather, they are emerging as fundamental regulators in a wide range of molecular biological processes, thereby defining a new and crucial layer of regulation in stem cell biology [8,9]. LncRNAs are broadly classified as transcripts longer than 200 nucleotides, typically transcribed by RNA polymerase II, spliced, and polyadenylated [10]. It is estimated that the human genome contains approximately 16,000 lncRNA genes, which can be further profiled into five distinct subgroups: (1) long intergenic non-coding RNAs (lincRNAs), which do not overlap with the annotated coding genes; (2) sense lncRNAs, which are transcribed from the sense strand of protein-coding genes and can even overlap with entire introns; (3) natural antisense transcripts (NATs), transcribed from the opposite strand of the protein-coding sense transcripts [11]; (4) enhancer RNAs, which stabilize the interaction between enhancers and promoters of nearby target genes [12]; and (5) transcribed ultra-conserved elements (T-UCEs), derived from DNA sequences and with the remarkable feature of retaining extended perfect sequence identity between human, mouse, and rat genomes [13]. LncRNAs are developmentally and temporally regulated by gene expression [14,15,16,17,18]. Since lncRNA expression is more cell- and tissue-specific than mRNA expression [19,20,21,22], stem cells offer a useful system for studying their critical functions during stem cell differentiation and somatic cell reprogramming [23]. The highly multifaceted nature of lncRNAs in exhibiting distinct cytotopic localizations (i.e., nucleus/cytosol) and in operating via different modes of action and with different target specificity is advancing the idea that they participate in many different regulatory pathways, reflecting their multi-functional role in cells [19,20,21,24]. By interplaying with different types of molecular factors, including DNA, RNA, and proteins, they generate an intricate network of interactions with transcripts, enhancers, and chromatin-modifier complexes [25]. This network endows lncRNAs with the capacity to transduce higher-order regulatory networks during stem cell differentiation by orchestrating the patterning of cells into tissues during development [13,26]. Although growing evidence indicates that lncRNAs exert critical physiological functions involved in genome integrity, cellular homeostasis, and metabolic responses in adult tissue stem cells, as well as in cell lineage commitment and the pluripotency network, their mode of action remains elusive and few have been mechanistically characterized to any great extent. To date, several annotated lncRNAs are preferentially localized in the nucleus, though very few have been functionally characterized as regulators of gene expression in different cellular contexts and events, including transcriptional and post-transcriptional regulation and reorganization of chromatin architecture. However, the biological role of several lncRNAs in vertebrate development such as *Hotair* [27], *MIAT*/*Gumafu* [28], *Evx1*-*as* [29], *Braveheart*, and *Haunt* [30] is still debated. Mutant studies in mouse models reported the dispensable role of some lncRNAs in viability, embryogenesis, and fertility, raising the possibility of their phenotypical redundancy and challenging the paradigm that RNA itself does not play a role in the observed functions [31,32]. Moreover, some lncRNAs were shown to exert a local regulation by acting as promoters for *cis*-regulation of neighboring genes [19,33]. Here, we summarize recent advances that have revealed the functions and molecular mechanisms involved in transcriptional regulation of annotated nuclear-retained lncRNA in stem cell biology, focusing on pluripotency and stem cell differentiation. This review addresses the principles of how lncRNAs cross-talk with chromatin by recruiting, tethering, and modulating the activity of epigenetic factors. In particular, we discuss the functional and intimate partnership between the chromatin modifier Polycomb repressive complex (PRC) 2 and lncRNAs as a classic example of how these molecules shape the epigenetic landscape and how this impacts on cell-fate programming and reprogramming. We also examine the latest insights into lncRNA functional roles, due in part to the advent of single-cell analysis, as well as future perspectives and current limitations in the field.

## 2. Nuclear-Retained LncRNAs and Their Functions

Among the well-studied lncRNAs, many reside in the nucleus where they play a variety of roles in basal cellular processes, including direct interaction with the transcription machinery, RNA splicing and maturation, chromatin remodeling, and the organization of nuclear domains [19,34]. LncRNAs perform these functions through different mechanisms: (1) They can act as transcriptional regulators with other nucleic acids forming DNA/RNA duplexes able to activate or repress transcription via cis-acting regulation of the expression of genes near their site of synthesis on the same chromosome, or trans-acting regulation of the expression of distally located genes across multiple chromosomes. LncRNAs can also interact directly with TFs, facilitating their localization and the association of TFs or other co-factors with chromatin in a cell- or tissue-specific manner [35,36]; (2) They participate in post-transcriptional events by modulating alternative splicing of sets of genes and controlling the stability of coding transcripts [37,38]; (3) They are involved in the organization of nuclear structure, interacting with the nuclear matrix either by acting as a ‘scaffold’ to modulate interchromosomal interactions or by rearranging nuclear speckles, structures involved in processing, maturation, and the export of precursor mRNA (pre-mRNA) [39,40]; (4) They regulate methylation and epigenetic states, guiding chromatin-remodeling factors to specific genomic loci by cooperating with histone readers, writers, and erasers—chromatin modifier enzymes that add, recognize, or remove post-translational modifications on histone proteins [41,42]. Among all the functions assigned to nuclear lncRNAs to date, epigenetic modifiers are the most frequent coupled protein partners [42]. Recent technologies probing lncRNA and chromatin interactions include RNA immunoprecipitation (RIP), cross-linking and immunoprecipitation (CLIP), chromatin isolation by RNA purification (ChIRP), capture hybridization analysis of RNA targets (CHART), and RNA antisense purification (RAP) [43]. These approaches, together with high-throughput tiling microarrays and innovations in next-generation sequencing (NGS)-based techniques applied to transcribed regions, have generated a genome-wide map of lncRNA-chromatin interactions providing direct evidence that a plethora of lncRNAs are associated with specific genomic regions, that they fold into domains, and that by recruiting epigenetic modulators, they mediate chromatin loop formation and control gene expression. Specifically, several lncRNAs were shown to interact with repressing and activating complexes, such as PRCs [43], involved in chromatin compaction and transcriptional silencing, and the SWitch/Sucrose NonFermentable (SWI/SNF) chromatin-remodeling complex [44]. In the following sections, we discuss the latest insights into lncRNA-mediated epigenetic regulations in the balance between ESC self-renewal and differentiation, and in particular, the modification of chromatin states via lncRNA-PRC2 interplay acting as a repressor of gene expression [44].

## 3. Polycomb Repressive Complex 2 in Embryonic Stem Cells

**Figure 1:**
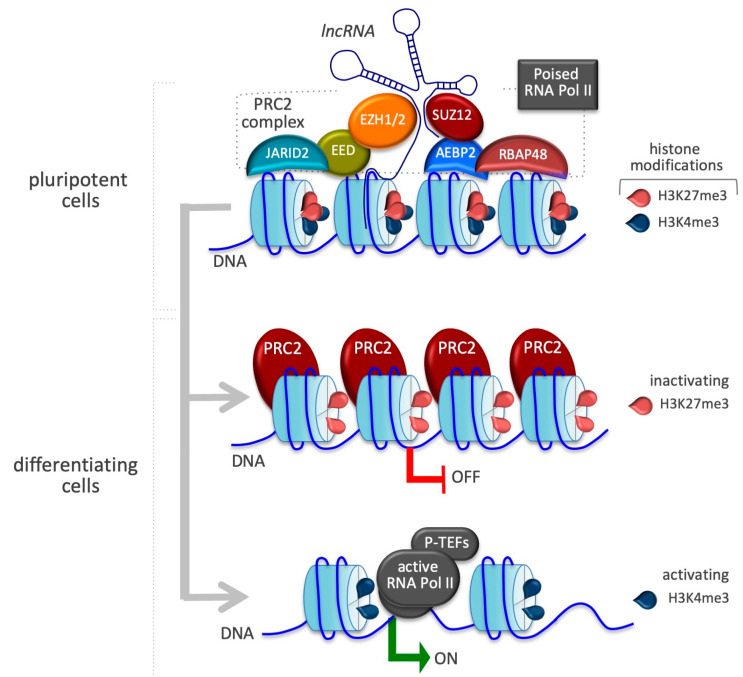
Pivotal role of PRC2 in stemness and differentiation. At bivalent domains, the simultaneous presence of repressive and active chromatin marks counterbalance each other. PRC2, guided and stabilized by the cooperation of a variety of lncRNAs, confers repression by H3K27me3 catalyzed by the subunit SUZ12. Many of these bivalently marked genes are bound by a non-processive form of RNA polymerase II (poised RNA Pol II). Upon differentiation, bivalent chromatin genes that remain inactive in differentiated cells lose the active H3K4me3 mark. By contrast, activated bivalent chromatin genes lose the repressive H3K27me3 mark due to the displacement of PRC2.

ESCs show the remarkable property of retaining pluripotency gene expression programs while enhancing cell fate transitions. Polycomb proteins, initially identified in *Drosophila melanogaster* as repressors of *Hox* homeotic genes, are evolutionary conserved from invertebrates to mammals. These proteins play a critical role in developmental pathways ensuring the proper activation of differentiation programs [45,46,47]. In fruit flies, Polycomb complexes are recruited to chromatin by conserved and extended 1 kb DNA domains called Polycomb response elements (PREs) through the activity of PRE-binding proteins [48]. PRE-like elements have not yet been defined in mammals. However, the function of, and the epigenetic mechanism by which PRCs act as repressor complexes—facilitating chromatin oligomerization that leads to transcriptional silencing via inhibition of chromatin remodeling activity—are evolutionarily conserved in eukaryotic cells [49]. Two specific complexes, PRC1 and PRC2, have been extensively studied [50,51]. Emerging evidence indicates that these distinct complexes can work both individually and synergistically by sharing common target genes in order to increase the level of gene repression. Specifically, PRC2 catalyzes histone H3 lysine 27 (H3K27) methylation and is thought to recruit PRC1 at the promoter level by working as a docking site for the PRC1, which catalyzes ubiquitylation of histone H2A on lysine 119 (H2AK119ub), thus maintaining transcriptional repression [51,52]. The mammalian PRC2 core complex contains four key proteins: embryonic ectoderm development (EED), suppressor of zeste 12 (SUZ12), RBAP48, and the catalytic enhancer of the zeste homolog 1/2 (EZH1/2) (Figure 1).

EZH1/2 contain a putative RNA-binding domain. These histone methyltransferases are responsible for the enzymatic activity of PRC2 by catalyzing mono-, di-, and trimethylation of H3K27 (H3K27me1, H3K27me2, and H3K27me3, respectively) [53,54,55]. PRC2 is tightly involved in the early steps of embryogenesis. Although EZH1/2 and EED knockout (KO) mice exhibit embryonic lethality after implantation, PRC2 deficiency seems to be dispensable in ESC self-renewal since it alters global H3K27me3 patterns without modifying the expression level of pluripotency-associated genes. Nevertheless, EZH2 KO ESCs are primed to spontaneous differentiation, leading to an increase in the expression of PRC2-targeted genes biased to differentiation and lineage commitment [56,57]. PRC2 is responsible for establishing cell type-specific gene expression programs by associating several regulatory subunits to the core complex. These accessory proteins, also required for a proper enzymatic activity or able to increase the affinity with a putative RNA binding, could directly influence and modify the interaction between PRC2 and chromatin and modulate the transcription of selected genes driving stem cell differentiation [58]. Metal response element binding transcription factor 2 (MTF2) and Jumonji AT-rich interactive domain 2 (JARID2), are newly identified regulatory subunits that interact with PRC2 in ESCs [59]. MTF2 depletion is accompanied by an increase in expression of the core pluripotent genes maintaining ESCs in an undifferentiated state, thereby preventing differentiation [60,61]. JARID2, a member of the Jumonji protein family of histone demethylases but without catalytic activity, plays an essential role during the early stages of development, interacting with and regulating PRC2 activity and facilitating its recruitment to promoters of target loci [62]. JARID2 expression is regulated by OCT4, which together with NANOG and SOX2 constitutes a triad of TFs crucial for ESC self-renewal and pluripotency, and is rapidly downregulated upon exposure to proper differentiation signals [63]. ESCs lacking JARID2 are prone to differentiate, enabling the cell response to signals of differentiation [64]. The unique property of ESCs that enables them to maintain a stable pluripotent phenotype while simultaneously remaining poised to differentiate upon the occurrence of external stimuli is finely tuned by chromatin-based mechanisms in which key developmental regulatory genes are dynamically marked in promoter regions by bivalent epigenetic signatures. Bivalent chromatin domains are genomic loci characterized by simultaneous occupancy of both H3K4me3 and H3K27me3 epigenetic modifications in the same region and were shown to endow mouse ESCs with a unique epigenetic landscape [65,66] (Figure 1). The balance between positive and negative histone modifications determines whether RNA polymerase II, pre-loaded and phosphorylated at serine 5 of its C-terminal domain on the bivalent region, is paused or released from pause, thus facilitating recruitment of the positive transcription elongation factor (P-TEFb) and preparing genes for rapid activation [67]. Emerging findings show that the repressive H3K27me3 mark is deposited exclusively by PRC2 and that it is stabilized by JARID2 interaction, which also protects it from the activity of H3K27 demethylases and is crucial in establishing the poised RNA polymerase II. Upon terminal differentiation, the bivalent chromatin domain could be converted into either a stable active (H3K4me3) or stable repressed (H3K27me3) state by the action of H3K27 or H3K4 demethylases, respectively [13,68] (Figure 1).

## 4. LncRNA-Directed Chromatin Regulation via PRC2 in Cell Fate

Recent breakthrough discoveries postulate a mechanistic model in which PRC2 identifies its site-specific target genes via regulatory RNAs, thus pointing to a functional interplay between non-coding RNA and chromatin modifiers [69,70]. RIP sequencing technology provided for the first time genome-wide profiles of non-coding RNAs including antisense, intergenic, and promoter-associated transcripts that directly interact with PRC2 in ESCs, putting forward the concept that nuclear lncRNAs are key players in chromatin epigenetic status regulation [43]. They act as cofactors to recruit Polycomb proteins to target loci prevalently via the EZH2 subunit, introducing epigenetic changes to chromatin, in order to direct stem cell patterning in tissues and organs during development, thus raising the question of how PRC2 discriminates between distinct RNAs in physiological contexts. In the following subsections, we describe some well-studied examples of lncRNAs that modulate chromatin structure and function in stem cells and differentiation by interplaying with PRC2.

### 4.1. X Inactive-Specific Transcript (Xist)

**Figure 2:**
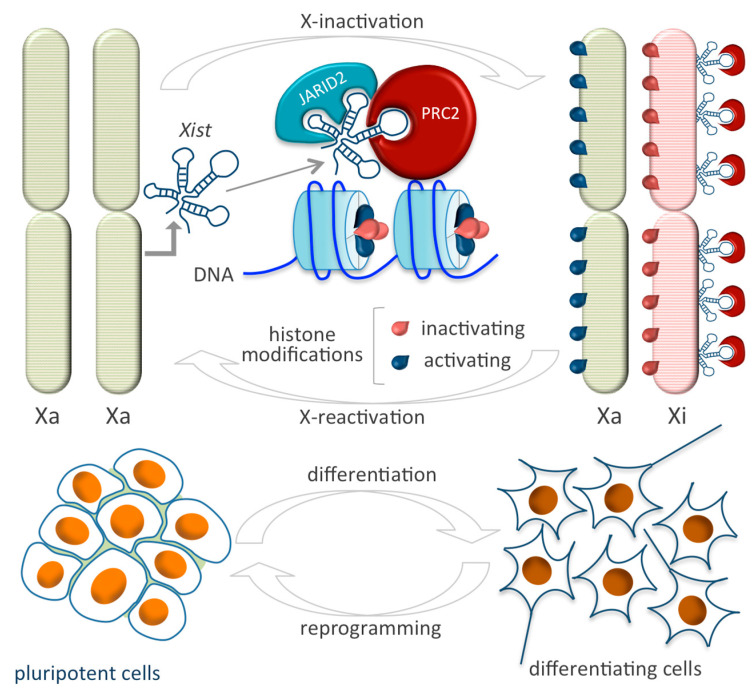
The X-chromosome state in females is linked to cell differentiation. Pluripotent cells have two active X-chromosomes (XaXa) and undergo X-chromosome inactivation when differentiated, resulting in one active and one inactive X-chromosome (XaXi). The lncRNA *Xist* is the main player in such process. The expression of the Xist gene on one of the X-chromosomes leads to the recruitment of the PRC2 complex to that chromosome, establishing its inactivation (**top** panel). During cell reprogramming, the X-chromosome state is reversed by X-chromosome Reactivation (**bottom** panel).

*Xist* was one of the first regulatory lncRNAs to be identified that challenged the simple recruitment model of epigenetic machinery to chromatin. This 17 kb lncRNA functions as a master regulator of chromatin epigenetic status and exemplifies the importance and versatility of lncRNAs in multiple physiological cellular processes [71,72]. In the last two decades, the study of *Xist* has provided a greater understanding of how lncRNAs guide histone modifiers to modulate epigenetic control of gene transcription and institute stable repressive chromatin states [73]. These insights led to the identification of novel interactors and new molecular mechanisms underlying this intricate partnership. In mammals, *Xist* is responsible for the entire inactivation of one X-chromosome in female somatic cells by forming a transcriptionally repressed heterochromatic structure (termed inactive X, Xi, or Barr body) in order to equalize X-chromosome dosages between the sexes. Transcribed from the X-inactivation center, *Xist* initiates, propagates, and maintains repression by physically coating the X-chromosome in cis, thus triggering extensive histone methylation and chromatin compaction via recruitment of chromatin modifiers [74,75,76,77] (Figure 2).

*Xist* levels are finely tuned by two nearby genes transcribed from the active X-chromosome, one of which is *Jpx*. The *Jpx* gene transcribes a lncRNA inducing *Xist* transcription and the antisense lncRNA *Tsix*, which enforces *Xist* silencing either by recruiting DNA methyltransferase 3a (*Dnmt3a*) on the *Xist* promoter or via RNA antisense targeting [78,79]. PRC2 is recruited for initiation of the X-chromosome inactivation (XCI) by direct and specific binding with *RepA*, a 1.6 kb lncRNA located within the 5′ end of the *Xist* gene spanning the evolutionary conserved A-repeat domain of *Xist* [80,81]. By RIP-seq, it was demonstrated that EZH2 and JARID 2 mediate the binding of PRC2 to *Xist* [43] (Table 1) and facilitate PRC2 recruitment on chromatin, which lays down the repressive H3K27me3 mark. *Tsix* hinders *RepA*-PRC2 function by titrating and preventing *RepA*-PRC2 transfer to chromatin and modulating PRC2 activity. *RepA* depletion abolishes full-length *Xist* induction and H3K27me3 deposition, while PRC2 silencing affects *Xist* upregulation [82,83]. Using an unbiased proteomic approach, a recent study identified an RNA-binding protein, ATRX, with high affinity for PRC2, which mediates direct binding of the epigenetic complex to *Xist*. ATRX silencing leads to spatial redistribution of PRC2 occupancy by altering Polycomb target gene regulation at the global level on the genome [84]. PRC1 is also involved in chromosome silencing induced by *Xist*. The noncanonical Polycomb group RING finger 3/5 (PCGF3/5)-PRC1 complex was shown to initiate recruitment of both PRC1 and PRC2 during XCI by catalyzing post-translational modifications of core histone proteins via mono-ubiquitylation of histone H2A on lysine 119 (H2AK119ub1) and H3K27me3, respectively. An RNA-binding protein is required to recruit PCGF3/5-PRC1 and guide binding with *Xist* via a specific *Xist*-Polycomb interaction domain, a 600 nucleotide sequence containing the *Xist* B-repeat element. PCGF3/5 depletion leads to female-specific embryo lethality and abolishes *Xist*-mediated gene repression [85]. Biallelic X-chromosome activation is considered an important hallmark of ESC pluripotency, as it is able to stabilize the naïve state of pluripotent stem cells by arresting differentiation. Indeed, the grade of pluripotency distinguishing the naïve and primed pluripotency of female ESCs and epiblast-derived stem cells, as well as the identification of fully reprogrammed colonies in induced pluripotent stem cell (iPSC) cultures, can be defined by the X-chromosome methylation status [86,87]. The presence of double X-chromosome activation inhibits MAPK and GSK3 signaling, known to be key pathways promoting the exit from the pluripotent state, and stimulates LIF/STAT3 and PI3K/AKT pathways required to maintain self-renewal and pluripotency. On adopting a specific cell fate, ESCs exit from pluripotency and the transition toward a differentiated state is characterized by dramatic chromatin changes leading to XCI. In this scenario, the germline factor PRDM14 exerts a critical function in self-renewal and ESC pluripotency by playing a dual role in repressing *Xist* during X-chromosome reactivation (XCR). PRDM14 silences the *Xist* activator Rnf12 via recruitment of PRC2 and directly targets *Xist* by binding intron 1. Gain-of-function experiments showed that PRMD14 overexpression accelerates XCR during the conversion of epiblast-derived stem cells to ESC transition and affects iPSC derivation and maintenance [88]. iPSC reprogramming is also associated with XCR, underscoring the need for chromatin remodeling to achieve a completely undifferentiated state. Female iPSCs are less stable in long-term culture compared to male-derived iPSCs and undergo erosion of dosage compensation, which compromises H3K27me3 foci in XCI and *Xist*, leading to ectopic expression of genes from the inactive X-chromosome [89]. As in *Xist*-mediated XCI, the 91 kb-long lncRNA *Kcnq1ot1*, exclusively localized in the nucleus, is important for bidirectional repression of genes in the *Kcnq1* domain [90,91]. *Kcnq1ot1* induces and maintains transcriptional silencing by recruiting the histone methyltransferase G9a and PRC2 via a lineage-specific mechanism. Specifically, its interaction with chromatin and subsequent repressive patterns were found predominantly in extraembryonic compared to other embryonic tissues. Within the *Kcnq1* domain, repressive histone marks span both nearby genes located within 200 kb of the *Kcnq1* imprinting control region and distantly located imprinted genes only in the placenta, underlying lineage-specific silencing modes of action [92].

### 4.2. Maternally Expressed 3 (Meg3)

*Meg3*, also known as gene trap locus 2, is a lncRNA encoded in the imprinted *Dlk1-Dio3* gene region on chromosome 14q32.2 [98,99]. The cis-elements regulating *Meg3* expression comprise two differentially methylated regions (DMRs): intergenic (IG)-DMR and *Meg3*-DMR [100]. *Meg3*, together with multiple non-coding RNAs and small non-coding RNAs, including the largest mammalian miRNA cluster, is expressed from the maternally inherited allele only. IG-DMR and *Meg3*-DMR are unmethylated on the maternal allele, whereas they are methylated on the paternally inherited allele, from which three protein-coding genes (*Dlk1*, *Rtl1*, *Dio3*) are expressed [100]. *Meg3* gene deletion obtained by targeting either *Meg3* or IG-DMR is embryonically lethal and different phenotypes are observed depending on the KO model [101,102,103]. Furthermore, the *Dlk1-Dio3* gene locus expression is linked to the optimal cellular reprogramming process and efficiency of the iPSC generation. RNA expression patterns of ESCs and iPSCs were in fact shown to be essentially indistinguishable with the exception of few maternally expressed non-coding transcripts originating from the *Dlk1-Dio3* cluster, which is silenced in the majority of iPSC lines, thus compromising the ability of cells to generate entirely iPSC-derived adult mice (known as ‘all-iPSC mice’) [104]. However, this remains a controversial issue, and further studies will be needed to fully define the tight correlation between the loss of imprinting at the *Dlk1-Dio3* locus and reduced pluripotency [105].

Mechanistic studies reported a role for *Meg3* in epigenetic regulation through its interaction with chromatin-modifying complexes such as PRC2, thereby guiding these complexes to genomic sites via DNA/RNA triplex formation [106]. The physical interaction between *Meg3* and EZH2 was described [93] (Table 1) and *Meg3* was shown to be indispensable for the proper recruitment and assembly of PRC2 in a subset of target genes in human iPSCs [94]. In addition, native and cross-linked RIP of JARID2 revealed that *Meg3*, as well as other lncRNAs deriving from the imprinted *Dlk1-Dio3* locus, interacts with PRC2 via JARID2 [94]. In the absence of JARID2, the interaction between *Meg3* and PRC2 is much weaker, suggesting that the contact point of JARID2 gives the major contribution to the affinity for *Meg3* [94]. However, in vitro binding analysis of *Meg3* suggests that JARID2 does not bind to RNA in a sequence-specific manner, in line with the finding that the primary sequences of lncRNAs are not generally well conserved [24,107].

Of note, although the mechanistic details of epigenetic alteration of the imprinted *Dlk1-Dio3* locus during reprogramming remain elusive, it is plausible that the interaction between *Meg3* and PRC2 plays an important role [108].

### 4.3. Trans-Spliced Rhabdomyosarcoma 2 Associated Transcript (tsRMST)

**Figure 3:**
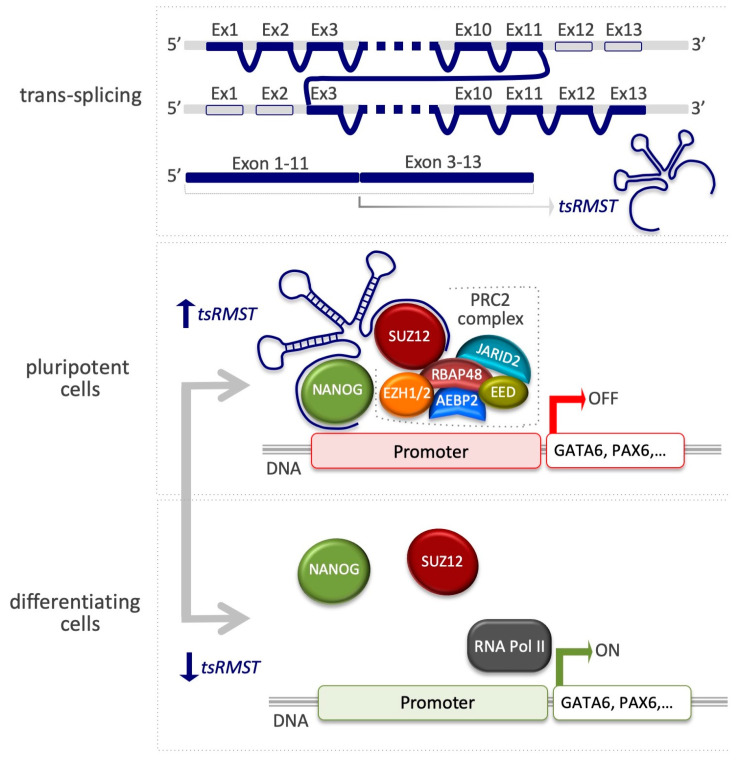
The lncRNA tsRMST suppresses lineage differentiation in hESCs. A scheme representing the exon order of tsRMST (upper panel). In pluripotent cells, the interaction of tsRMST with NANOG and the PRC2 complex promotes occupancy on inactive genes, such as GATA6 and PAX6 (middle panel). In differentiating cells, the absence of tsRMST leads to the displacement of PRC2 with the consequent induction of the lineage-specific genes (bottom panel) [95].

Alternative splicing, which arises from post-transcriptional events, can lead to the generation of multiple transcript isoforms from a single gene, thus providing an essential source of diversity for the transcriptome and proteome [109,110,111,112,113]. Splicing can occur either in cis or in trans [114,115]. A single pre-mRNA is originated by *cis*-splicing of exons, whereas two or more separate pre-mRNAs are derived either from the same gene or from two or more different genes by intragenic and intergenic trans-splicing, respectively. Trans-splicing is largely detected by comparing reference genomes with expressed sequence tags (ESTs)/mRNAs [116,117,118,119] or by the NGS of mRNAs (RNA-seq) [120,121,122,123]. However, these kinds of approaches generate a significant number of false positives. To tackle this issue, a computational pipeline named TSscan, based on transcriptome sequencing data from different NGS platforms and several undifferentiated hESC lines was developed, and confirmed that four trans-splicing events (*tsCSNK1G3*, *tsARHGAP5*, *tsFAT1*, *tsRMST*) occur in hESCs [95]. All four trans-spliced RNAs are also expressed in human iPSCs, suggesting that these events tend to occur in human pluripotent stem cells. Of particular interest is *tsRMST*, the first trans-spliced lincRNA to be identified, which is highly expressed in both hESCs and iPSCs, and whose expression is reduced after in vitro hESC differentiation, suggesting the possibility that *tsRMST* is specifically expressed in pluripotent stem cells, and may thus play a role in pluripotency maintenance. Microarray-based genome-wide expression profiling performed on *tsRMST* knockdown hESCs showed that the expression levels of pluripotency-associated genes such as *NANOG*, *POU5F1*, and *SOX2* were significantly decreased, whereas key lineage-specific TFs such as GATA6 (endoderm) and PAX6 (neuroectoderm) were induced [95]. At the mechanistic level, RIP analysis showed that *tsRMST* interacts directly with SUZ12 (Figure 3 and Table 1) and NANOG. In line with these results, chromatin immunoprecipitation qPCR experiments on *tsRMST* promoters repressing lineage-specific genes (*GATA6* and *PAX6*) further showed the loss of NANOG and SUZ12 occupancy, as well as H3K27me3 modification in *tsRMST* knockdown hESCs (Figure 3) [95]. A model was proposed in which *tsRMST* suppresses lineage differentiation in hESCs via recruitment of NANOG and PRC2 [95] and plays a regulatory role in noncanonical Wnt signaling [124].

### 4.4. Long Intergenic Noncoding HOXA1 (Linc-HOXA1)

**Figure 4:**
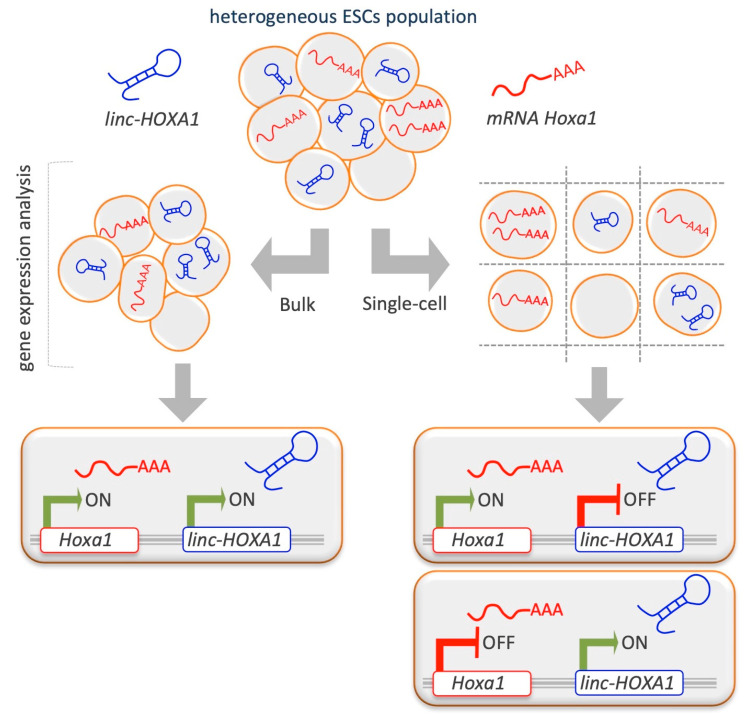
Comparison of single-cell versus bulk analysis results in lncRNA studies. ESCs show a cellular heterogeneity of *linc-HOXA1* and *Hoxa1* RNA within the population. A single-cell gene expression analysis highlighted an inverse correlation between *linc-HOXA1* RNA and *Hoxa1* mRNA abundance. Before the advent of the single-cell approach, the bulk analysis were showing an average result, losing the real feature of cells and masking the close interaction between *linc-HOXA1* and *Hoxa1* [96,125].

*Linc-HOXA1* (also known as *Haunt, Halr1*, and *Gm15055*) is located approximately 50 kb downstream of the *Hoxa* gene cluster. By single-cell transcript counting performed in mESCs, there emerged a considerable heterogeneity of *linc-HOXA1* and *Hoxa1* RNA in pluripotent cultures at single-cell resolution and an inverse correlation between *linc-HOXA1* RNA and Hoxa1 mRNA abundance (Figure 4) [96]. Single-cell multiplex transcript counting analysis is crucial to see the effects that, as will be discussed in ‘State of the Art and Future Perspectives’, might be hard to observe using bulk measurements.

At the molecular level, *linc-HOXA1* recruits the protein PURB as a transcriptional cofactor, thereby repressing *Hoxa1* transcription in cis [125]. PRC2 is recruited to the *Hoxa* locus and is part of a complex mechanism for fine-tuning *Hoxa* gene regulation in mESCs. The *linc-HOXA1* gene locus contains an OCT4-responsive positive cis-regulatory element, which potentially regulates the lincRNA itself and *Hoxa* gene activation. Further studies based on the generation of *linc-HOXA1* KO ESCs, aimed at investigating the regulation of *Hoxa* gene expression and chromatin status in mESCs, highlighted by RIP the interaction between *linc-HOXA1* and PRC2 [125]. Specifically, *linc-HOXA1* recruits PRC2 to the *Hoxa* gene cluster to maintain the repressive histone mark H3K27me3 on *Hoxa* gene promoters in mESCs. Alkaline phosphatase (a stem cell membrane marker) staining, as well as immunofluorescence and RT-qPCR analysis of the pluripotency markers SSEA1 and OCT4, confirmed that pluripotency of mESCs was not disrupted by *linc-HOXA1* knockdown, and excluded the possibility that the increased *Hoxa* gene expression was due to mESC differentiation [96]. In contrast, H3K27me3 modification and SUZ12 binding (Table 1) were decreased at the *Hoxa* gene promoters. However, no significant changes in H3K4me3 were observed at *Hoxa* gene promoters in KO ESCs [96].

### 4.5. The Transcribed Ultraconserved LncRNA T-UCstem1

The ultraconserved lncRNA *T-UCstem1* was recently identified and its role in ESC self-renewal maintenance was elucidated. *T-UCstem1* exerts a dual but distinct function in the nucleus and in the cytosol. In the nucleus, as demonstrated by RIP, *T-UCstem1* directly binds PRC2, recruiting the complex to bivalent chromatin-associated genes. In *T-UCstem1-*depleted cells, PRC2 displacement leads to an increased H3K4me3/H3K27me3 ratio at bivalent chromatin domains, committing ESCs to exit from pluripotency and to differentiation [13]. Interestingly, absence of *T-UCstem1* determines a cellular phenotype similar to that resulting from the withdrawal of PRC2 activity from ESCs, and a global gene derepression of bivalent chromatin-associated genes (Table 1) leading to spontaneous differentiation [67,126,127].

### 4.6. Heart-Associated LncRNA Braveheart (Bvht)

*Bvht* is a heart-associated lncRNA in the mouse. Using multiple ESC differentiation strategies, *Bvht* was shown to be essential for nascent mesoderm progression towards cardiac fate. Specifically, *Bvht* triggers a cardiovascular gene network core upstream the mesoderm posterior 1 (*MesP1*) (Table 1), a master regulator of multipotent cardiovascular progenitors. At the molecular level, by native RIP, *Bvht* was shown to interact with SUZ12 at numerous stages during ESC-cardiac differentiation directly. In cells lacking *Bvht* expression, SUZ12 and the repressive modification H3K27me3 are enriched at promoters of cardiac-associated genes, such as *MesP1*. Interestingly, these genes still exhibit bivalent chromatin domains in *Bvht*-depleted cells, similar to their initial configuration in ESCs, in line with the inability of these cells to trigger cardiac cell commitment [97]. Of note, *Bvht* is an example of lncRNA involved in differentiation while all the others we mentioned play a role in the stemness maintenance.

Of note, LncRNAs can also influence the interaction between PRC2 and other epigenetic modifying complexes, working as a scaffold to bind multiple chromatin-modifier structures. For instance, the lateral mesoderm-specific lncRNA *Fendrr*, critical for cardiac mesoderm specification, positively modulates chromatin state to activate a subset of developmental regulators by binding both PRC2 and Trithorax group/MLL protein complexes (TrxG/MLL) [128]. Recent studies also described lncRNA binding in the regulation of active chromatin configuration states. TrxG/MLL, which catalyzes methylation of the activation epigenetic mark histone H3 at lysine 4, could be recruited to the HoxA promoter by the lncRNAs *HOTTIP* and *Mistral* through the interaction with WDR5, a specific subunit of the MLL complex, or the lncRNA *HoxBlinc*, which is associated with active chromatin states by regulating cardiac/hematopoietic differentiation [129].

## 5. State of the Art and Future Perspectives

LncRNAs participate in a wide variety of stem cells and developmental physiological processes such as transcriptional regulation, chromatin modification, imprinting, DNA methylation, and splicing, introducing a novel layer of biological regulation. The functional role of lncRNAs in chromatin remodeling in a temporal and cell type-specific manner has become a hot topic. LncRNAs act as important cofactors in mediating PRC2 recruitment to specific target loci, thus determining specific chromatin states corresponding to different stages of embryonic development and stem cell differentiation. Although promiscuous RNA binding to PRC2 is described [83], several studies underscore the importance of lncRNA binding to this chromatin remodeling complex, which then mediates cofactor recruitment conferring its specificity of action. While ESCs have provided a powerful model for investigating the developmental role of these multifaceted molecules, research into the molecular mechanism by which lncRNAs guide PRC2 to chromatin is still in its infancy and current knowledge is greatly limited by methodological shortcomings. In addition, the extremely cell-specific mode of action of lncRNAs makes the understanding of their molecular mechanisms even more challenging.

### 5.1. Current and Future Methodologies

Several methods have recently been developed to map lncRNAs on chromatin, shedding considerable light on their biological importance in epigenetic regulation. Similar to the well-established ChIP technique, RIP has proved to be a powerful tool to study the direct binding between individual proteins and RNA molecules in close proximity [130,131]. Using a specific antibody against the protein of interest, the RNA-binding protein is pulled down and further analyzed by PCR-based approaches, hybridization, or sequencing. RIP can be carried out either in a native condition to identify lncRNAs directly associated with the protein and to quantify their abundance within immunoprecipitates, or by using CLIP strategies where whole tissues or cells are treated with UV-B irradiation. Cross-linking generates a covalent bond, which serves to precisely map the direct and indirect binding sites between RNA-protein complexes. Recent technologies directly probing lncRNAs have greatly advanced our understanding of lncRNA function in ESC gene regulation. CHART is able to identify where lncRNAs localize, as well as their protein partners, providing a genomic map of lncRNA targets [130]. The biotinylated complementary oligonucleotides are designed to have a high affinity for the lncRNA of interest. Similarly, RAP identifies regions of the target RNA through hybrid capture via antisense oligonucleotides, allowing sequencing of RNA and DNA from purification samples, but like CHART, requires prior knowledge of the lncRNA sequence of interest. ChIRP is another useful and rapid method to study the role of lncRNA in epigenetic regulation. ChIRP maps genomic binding sites of chromatin-associated lncRNAs, identifying lncRNA-chromatin interactions by including complementary oligonucleotides that hybridize to the target lncRNA [130]. All these techniques can be extended genome-wide using sequencing methodologies and integrated to identify the genomic localization of RNA and its coupled protein, generating a more comprehensive profile of lncRNA function, including recruitment of chromatin-modifiers.

### 5.2. The Advent of Single-Cell Technologies: Limits and Potential

Nascent NGS technologies at single-cell resolution could provide valuable insights into the complex role of lncRNA in stem cell differentiation. Single-cell RNA sequencing (scRNAseq) correlates the transcriptional profile of individual cells to the complexity of biological systems within a population [132,133,134]. ScRNAseq provides a better understanding of the function of a single cell within the context of its microenvironment by identifying novel cell types and characterizing heterogeneous subtypes (Figure 3), highlighting regulatory relationships between coding and non-coding genes, and tracing the trajectories of distinct cell lineages during differentiation. High-resolution at the single-cell level could overcome the current limitation in bulk RNA sequencing due to the low abundance of lncRNAs and the difficulty in distinguishing them from transcriptional noise. A recent study reported the direct comparison of lncRNA profiles by combining bulk tissue sequencing and single-cell RNAseq of the human neocortex at different stages of development. Single-cell transcriptomic analysis provided high-resolution data revealing that lncRNAs generally detected at low levels in bulk-seq are in fact abundantly expressed in individual cells, showing remarkable cell-type specificity. This may also explain why some lncRNAs display reduced expression in tissues [135]. Single-cell sequencing also addresses another major hurdle related to the tight connection between lncRNAs and the biological milieu in which they function [96]. However, precisely, how transcriptional heterogeneity and changes in the transcriptome reflect pluripotent states and differentiation can only be clarified by investigating the epigenome at the single-cell level. Since transcriptional changes precede or follow epigenetic marks, single-cell epigenome sequencing provides a high-resolution map of chromatin accessibility, including open or closed chromatin states and nucleosome positioning. Taken alone, scRNAseq results leave unanswered questions that might be solved by integrating multiple levels of information. For instance, to better unravel the molecular mechanisms underlying lncRNAs and their role in tethering the PRC2 complex and other chromatin modifiers, an integrative analysis combining ChIPseq and scRNAseq experiments could be useful. Despite being a challenging task, the possibility of obtaining an integrated analysis of multi-omic profiles at the single-cell level would undoubtedly be a powerful tool. Combined single-cell RNA and epigenome sequencing could break up intricate regulatory systems into individual layers, providing new insights into how genetic variation is related to transcriptional variation. The technological advancements for efficiently identifying open chromatin regions and regulatory elements across the genome are ongoing. A variety of assays have been designed to assess how chromatin compaction and DNA-binding proteins regulate gene expression at single-cell resolution since epigenetic regulation comes in different forms. Nevertheless, epigenome-based methods have several limitations when used at the single-cell level, yielding lower throughput and scalability compared to scRNAseq.

Currently, the use of bisulfite sequencing to study nucleosome occupancy and methylome at the single-cell level limits the capture rates and does not provide extensive and functional mapping of chromatin states at single-cell resolution. In contrast, the assay for transposase-accessible chromatin sequencing (ATAC-seq) will likely become a routinely used strategy in single-cell analysis and represents an important step forward in assessing epigenetic diversity. ATAC-seq identifies genome-wide chromatin accessible regions by using a transposase enzyme to insert and tag open chromatin. Via a microfluidic approach, this technique uses barcoding to successfully process tens of thousands of single cells in parallel, providing a regulatory landscape of the genome at the epigenetic level. A common drawback in both single-cell transcriptomics and epigenomics is technical variability. Although the level of technical noise in scRNAseq could be estimated and normalized, such strategies have not yet been developed in epigenome sequencing [136].

## 6. Conclusions

Their extremely tissue-specific expression and exclusive mode of action further complicate our understanding of the role of lncRNA in physiological processes. Ongoing studies are, therefore, combining genome-wide transcriptional analysis of single cells with the complexity and heterogeneity of tissue structures by using in situ transcriptomics technology to map lncRNAs based on their localization. Thanks to their cell type- and organ-specific expression patterns, lncRNAs might be employed as candidate targets for stem cell-based therapies and other clinical applications such as the screening of new drugs to introduce chromatin changes. While epigenetic drugs produced to date function mainly as enzyme inhibitors by attenuating their activity and inducing drastic chromatin changes in the genome, manipulating lncRNA expression that normally targets one gene or a small set of genes may reduce the aberrant expression of off-target genes and facilitate the development of small-molecule drugs with more specific modes of action. Loss-of-function studies using both small interfering RNA and antisense oligonucleotides, as well as gain-of-function experiments describe only local alterations without detecting changes in overall chromatin configuration. Moreover, deciphering the ‘epigenetic code’ of lncRNA could unravel the intricate network of these different molecule species, elucidating the molecular mechanisms controlling stem cell differentiation and their potential involvement in tissue regeneration.

## Figures and Tables

**Table 1:** PRC2 protein partners and the regulatory function of lncRNAs.

LncRNAs	PRC2 Protein Partners	Regulatory Function	Refs.
*Xist*	EZH2, JARID 2	X-chromosome inactivation	[82]
*Kcnq1ot1*	EZH2, SUZ12	*Kcnq1* inactivation	[92]
*Meg3*	EZH2, JARID2	ESC subset of genes inactivation	[93,94]
*tsRMST*	SUZ12	Pluripotency-associated genes activation (*NANOG*, *POU5F1*, *SOX2*) and cell commitment genes inhibition (*GATA6*, *PAX6*)	[95]
*Linc-HOXA1*	SUZ12	*Hoxa* inactivation	[96]
*T-UCstem1*	SUZ12	Global de-repression of bivalent chromatin-associated genes	[13]
*Bvht*	SUZ12	*Mesp1* activation	[97]
